# let-7i inhibits proliferation and migration of bladder cancer cells by targeting HMGA1

**DOI:** 10.1186/s12894-019-0485-1

**Published:** 2019-06-13

**Authors:** M-M Qin, X. Chai, H-B Huang, G. Feng, X-N Li, J. Zhang, R. Zheng, X-C Liu, C. Pu

**Affiliations:** 1 Clinical Laboratory, The First Affiliated Hospital of Wannan Medical College, No.2, West Zheshan Road, Wuhu, 241001 Anhui China; 2 Department of Urology, The First Affiliated Hospital of Wannan Medical College, Wuhu, 241001 Anhui China

**Keywords:** Let-7i, High mobility group protein A1, Bladder cancer, Proliferation, Migration

## Abstract

**Background:**

Let-7 is one of the earliest discovered microRNAs(miRNAs) and has been reported to be down-regulated in multiple malignant tumors. The effects and molecular mechanisms of let-7i in bladder cancer are still unclear. This study was to investigate the effects and potential mechanisms of let-7i on bladder cancer cells.

**Methods:**

Total RNA was extracted from bladder cancer cell lines. The expression levels of let-7i and HMGA1 were examined by quantitative real-time PCR. Cell viability was detected using the CCK-8 and colony formation assays, while transwell and wound healing assays were used to evaluate migration ability. Luciferase reporter assay and western blot were used to confirm the target gene of let-7i.

**Results:**

Compared with the SV-40 immortalized human uroepithelial cell line (SV-HUC-1), bladder cancer cell lines T24 and 5637 had low levels of let-7i expression, but high levels of high mobility group protein A1 (HMGA1) expression. Transfection of cell lines T24 and 5637 with let-7i mimic suppressed cell proliferation and migration. Luciferase reporter assay confirmed HMGA1 may be one of the target genes of let-7i-5p. Protein and mRNA expression of HMGA1 was significantly downregulated in let-7i mimic transfected cell lines T24 and 5637.

**Conclusions:**

Up-regulation of let-7i suppressed proliferation and migration of the human bladder cancer cell lines T24 and 5637 by targeting HMGA1. These findings suggest that let-7i might be considered as a novel therapeutic target for bladder cancer.

## Background

Bladder cancer represents a common malignancy in urinary system, and its morbidity tends to yearly grow. Over 400,000 new cases of bladder cancer are reported worldwide every year [1]. The statistics of National Cancer Institute of China estimated that new cases of bladder cancer were 80,500, and death of bladder cancer were 32,900 in 2015 in China [2]. Approximately 70–75% of newly diagnosed bladder cancers are non-muscle invasive (NMIBC) [3]. NMIBC is typically treated with endoscopic transurethral resection (TUR). However, most patients have high risk of recurrence and disease progression. The median survival of patients with bladder cancer was 15 months, and the 5-year survival rate of bladder cancer was only 15% [4]. Therefore, the molecular mechanism that regulates the progression of bladder cancer is of great significance for treatment of bladder cancer.

MicroRNAs (miRNAs) are endogenous, non-coding RNAs and they play an important regulatory role through complimentary binding of the 3′ untranslated regions (3’UTRs) of target genes in RNA silencing and post-transcriptional regulation of gene expression [5]. Since the discovery of miRNAs [6], a variety of abnormal expressions of miRNAs have been found in many human cancers, including gastric cancer [7], breast cancer [8] and bladder cancer [9]. Abnormal expression of miRNAs and the growth, metastasis and apoptosis of human tumor cells were closely related [10–12]. Let-7 family is the earliest discovered miRNAs. Let-7 family play a significant role in the development and progression of many cancers, including prostate cancer [13] and bladder cancer [14]. However, the effects of let-7i, a member of the let-7 family, in bladder cancer are still unclear.

High mobility group protein A1(HMGA1), a member of the HMGA family, can form multi protein stereo complexes by binding to the DNA region containing rich AT basic group, and regulate gene transcription of many genes [15]. HMGA1 as a key regulator of the autophagic pathway in cancer cells could contribute to cancer progression [16]. Previous study reported that let-7i was a key factor in development of prostate cancer by regulate HMGA2 [17]. Liu [18] also found that down-regulation of let-7a could inhibits growth and migration of breast cancer cells by targeting HMGA1.

However, how let-7i affects HMGA1 gene expression in bladder cancer cells are still unclear. Therefore, the aim of this study was to investigate the effects of let-7i on human bladder cancer cells proliferation and metastasis. Furthermore, to explore how let-7i affects HMGA1 expression in bladder cancer cells.

## Methods

### Materials

SV-HUC-1 and human bladder cancer cell lines T24 and 5637 were purchased from the Chinese Academy of Sciences (Shanghai, China); The 5637 series of bladder cancer is a human origin, which is a situ bladder cancer cell derived from the upper skin, with a moderate degree of malignancy. Bladder cancer T24, derived from human bladder transitional cell carcinoma cells, is an epithelioid metastatic adenocarcinoma with a high degree of malignancy. RPMI-1640 medium and fetal bovine serum were purchased from GIBCO (USA); CCK-8 Cell Proliferation Detection Kit was purchased from KeyGEN Bio TECH (Nanjing, China); 24-well plates with a transwell chamber was purchased from Corning (NY, USA); let-7i mimic, let-7i mimic negative control, HMGA1 primers and let-7i primers were purchased from RiboBio (Guangzhou, China); Trizol Universal reagent, miRcute Plus miRNA First-Strand cDNA Synthesis Kit and miRcute Plus miRNA qPCR Detection Kit (SYBR Green) were purchased from TIANGEN (Beijing, China); RevertAid™ First Strand cDNA Synthesis Kit was purchased from Thermo (Shanghai, China); SYBR®Premix Ex TaqTM(Tli RNaseH Plus) was purchased from TaKaRa (Beijing, China); The rabbit polyclonal antibodies against HMGA1 was purchased from Abcam (Cambridge, UK); The rabbit polyclonal antibodies against β-Actin was purchased from Cell Signaling Technology (Danvers, MA, USA).

### Cell culture and transfection

T24 and 5637 cells in this experiment were newly resuscitated cells, which underwent 7–8 biological replications and were tested after the cells were in a stable state. The cells were routinely cultured in the RPMI-1640 medium containing 10% fetal bovine serum, 100 U/mL of streptomycin, and 100 U/mL of penicillin in a humidified cell incubator. Cell lines T24 and 5637 were plated at a density of 2 × 10^5^ cells/well in 6-well plates. The let-7i mimic was cloned to Lipofectamine 3000, which was then transfected. Transient transfection was conducted using Lipofectamine 3000 (Invitrogen, USA) according to manufacturer’s instructions. The let-7i mimic and negative control are designed by RiboBio(Guangzhou, China). Let-7i mimic was mature miRNA. Let-7i mimic and negative control were used at a concentration of 100 nmol.

### Cell proliferation assay

Cell lines T24 and 5637 transfected with let-7i mimic or negative control were plated on 96-well plates at 2000 cells/well. After 24, 48 and 72 h of transfection, cells were incubated in 10% CCK-8 diluted in culture media at 37 °C until visual color conversion appeared. The absorbance was measured at 450 nm using a microplate reader according to the manufacturer’s protocol.

### Colony forming assay

Cell lines T24 and 5637 transfected with let-7i mimic or negative control were plated on 6-well plates at 500 cells/well and incubated in 5% CO_2_ atmosphere at 37 °C for 14 days. Fresh medium is changed every 3 days during this period. Then cells were fixed and stained, followed by colony counting.

### Wound healing and cell transwell assays

Wound healing and cell transwell assays were performed as previously described [18].

### RNA extraction and RT-PCR

Total RNA was extracted from the bladder cancer cell lines using Trizol Universal reagent according to the manufacturer’s instructions. Then, cDNA was obtained using the corresponding reverse transcriber reagent according to the manufacturer’s instructions in United States labnet PCR instrument (BIO-RAD, USA). Quantitative polymerase chain reaction (PCR) was performed in ABI 7500 Sequence Detection System (Life Technologies, USA) using the corresponding PCR reagent according to the manufacturer’s instructions. miRNA quantification with Bulge -loopTM miRNA RT-qPCR Primer Sets (one RT primer and a pair of qPCR primers for each set) specific U6 and let-7i are designed by RiboBio (Guangzhou, China). HMGA1 forward primer sequences: 5′-TCCATTCTTCGACATCCGTCA-3′ HMGA1 reverse primer sequences: 5′-GATCGTGGGCAGAACAGGAG-3′; GAPDH forward primer sequences: 5′-CATCAAGAAGGTGGTGAAGCAG-3′; GAPDH reverse primer sequences: 5′-GTGTCGCTGTTGAA.

GTCAGAG-3′. The relative quantification was performed by normalizing against the levels of GAPDH for mRNA or U6 for miRNA. Relative quantification of mRNA and miRNA expression was calculated using the 2^-△△Ct^ method.

### Protein extraction and western blot

Total protein was collected and lysed in 1 × Laemmli sample buffer (Sigma, USA) on ice; After the lysate boiled, protein samples (5 μl) were fractionated in 10% SDS-polyacrylamide gel and then transferred to nitrocellulose (NC) membrane (GE Healthcare, Piscataway, NJ, USA). Membranes were blocked with 10% non-fat milk and washed with TBST, and then incubated with primary antibody (dilution at 1:1000) at 4 °C for 12 h. The NC membranes were extensively washed three times, and then incubated with anti-rabbit horseradish peroxidase-conjugated secondary antibody. Following removal of the secondary antibody, the membranes were scanned by Fluor Chem FC3 (Protein Simple, San Jose, CA, USA). β-Actin was used as an internal control.

### Fluorescent reporter assay

Cells were cultured in 24-well plates and then co-transfected with 100 ng of HMGA1-UTR-WT or -MUT psi-CHECK2 vectors plus 100 nM let-7i-5p mimic or scrambled sequences using Lipofectamine 3000. 48 h after transfection, luciferase activities were measured using Dual-Luciferase Reporter Assay System (Promega, USA). Firefly luciferase activity was used as an internal reference standard.

### Statistical analysis

All statistical analyses were performed using the SPSS 19.0 version (SPSS Inc., Chicago, IL, USA) and GraphPad 5.0 software. Each experiment was performed in triplicate. All values for experimental results are expressed as the mean ± SEM. The statistical significance of differences between independent groups was determined by one-way analysis of variance (ANOVA) or t-test. A two-sided *P* value < 0.05 was considered statistically significant.

## Results

### Let-7i was down-regulated in bladder cancer cell lines

**Fig. 1:**
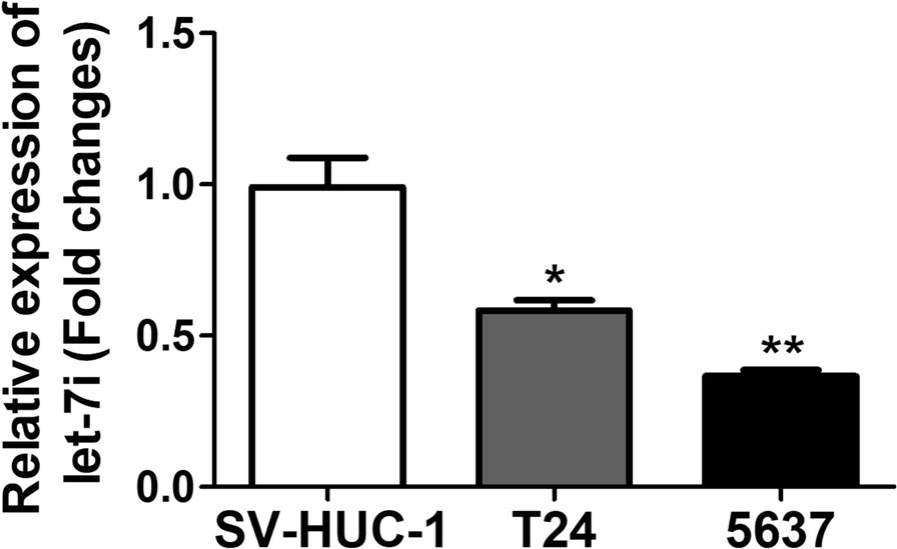
Let-7i expression levels in bladder cancer cell lines and SV-HUC-1

As shown in Fig. 1. Low expression of let-7i was found in bladder cancer cell lines T24 (0.58 ± 0.03) and 5637 (0.37 ± 0.02) as compared to SV-HUC-1 (0.99 ± 0.10, *P* < 0.05).

### Overexpression of let-7i inhibited bladder cancer cells proliferation

**Fig. 2:**
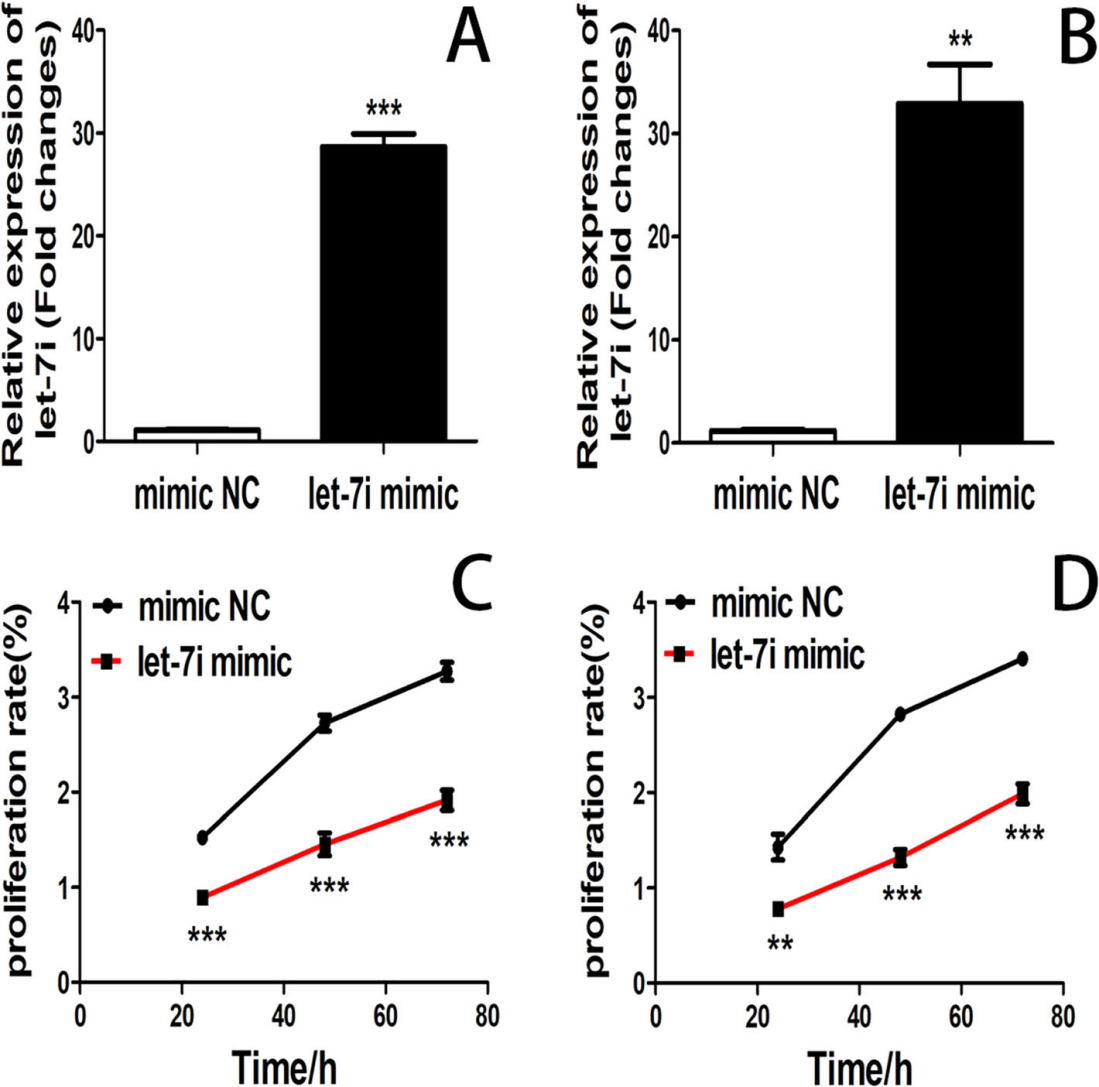
(**a**) The levels of let-7i expression in cell lines T24 transfected with let-7i mimic and negative control was detected by RT-PCR after transfection for 24 h. (**b**) The levels of let-7i expression in cell lines 5637 transfected with let-7i mimic and negative control was detected by RT-PCR after transfection for 24 h. (**c**) CCK-8 assay was used to determine the proliferation of cell lines T24 transfected with let-7i mimic and negative control. (**d**) CCK-8 assay was used to determine the proliferation of cell lines 5637 transfected with let-7i mimic and negative control. (**P < 0.01, ***P < 0.001)

Let-7i expression was effectively up-regulated in bladder cancer cell lines T24 and 5637 after transfected with let-7i mimic (Fig. 2a and b). CCK-8 assay was performed to detect the proliferation of cell lines T24 and 5637 after transfection with let-7i mimic for 24, 48, and 72 h. Overexpression of let-7i significantly inhibited cell proliferation compared with the negative control cells. (Fig. 2c and d).

### Overexpression of let-7i inhibited bladder cancer cells colony formation

**Fig. 3:**
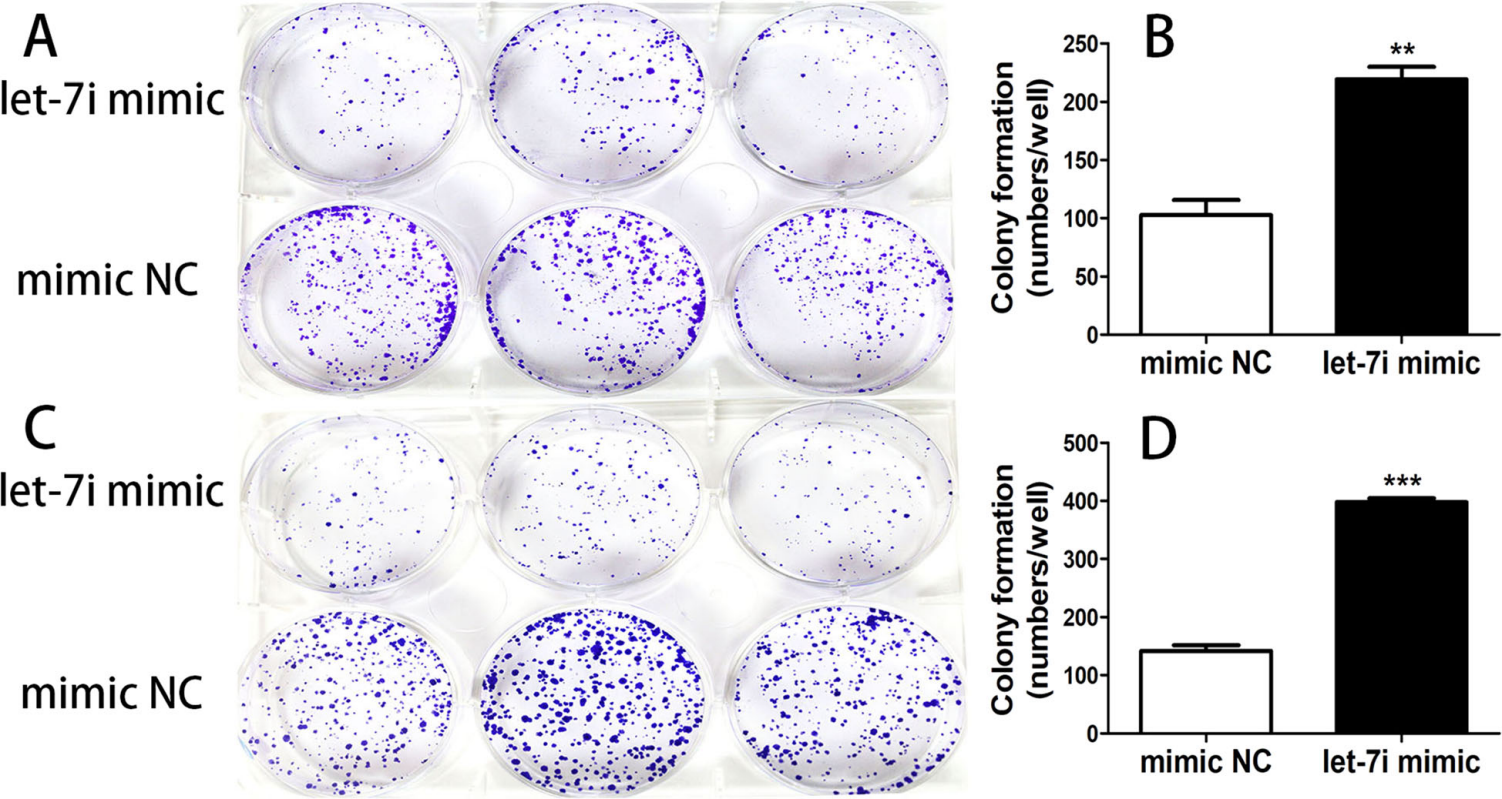
(**a**) The colonies of cell lines T24 transfected with let-7i mimic and negative control were stained by crystal violet at day 14 post-transfection. (**b**) The graph represents the mean of colony number ± SEM in cell lines T24 transfected with let-7i mimic and negative control. (**c**) The colonies of cell lines 5637 transfected with let-7i mimic and negative control were stained by crystal violet at day 14 post-transfection. (**d**) The graph represents the mean of colony number ± SEM in cell lines 5637 transfected with let-7i mimic and negative control. (**P < 0.01, ***P < 0.001)

Clone formation experiment was performed to detect the proliferation of cell lines T24 and 5637 after transfection with let-7i mimic and negative control for 14 days. Colonies formed from T24 cells transfected with let-7i mimic were significantly less than that of negative control transfected cells (Fig. 3a and b). While colonies formed from 5637 cells transfected with let-7i mimic were also significantly less than that of negative control transfected cells (Fig. 3c and d).

### Overexpression of let-7i suppressed T24 and 5637 cells migration

**Fig. 4:**
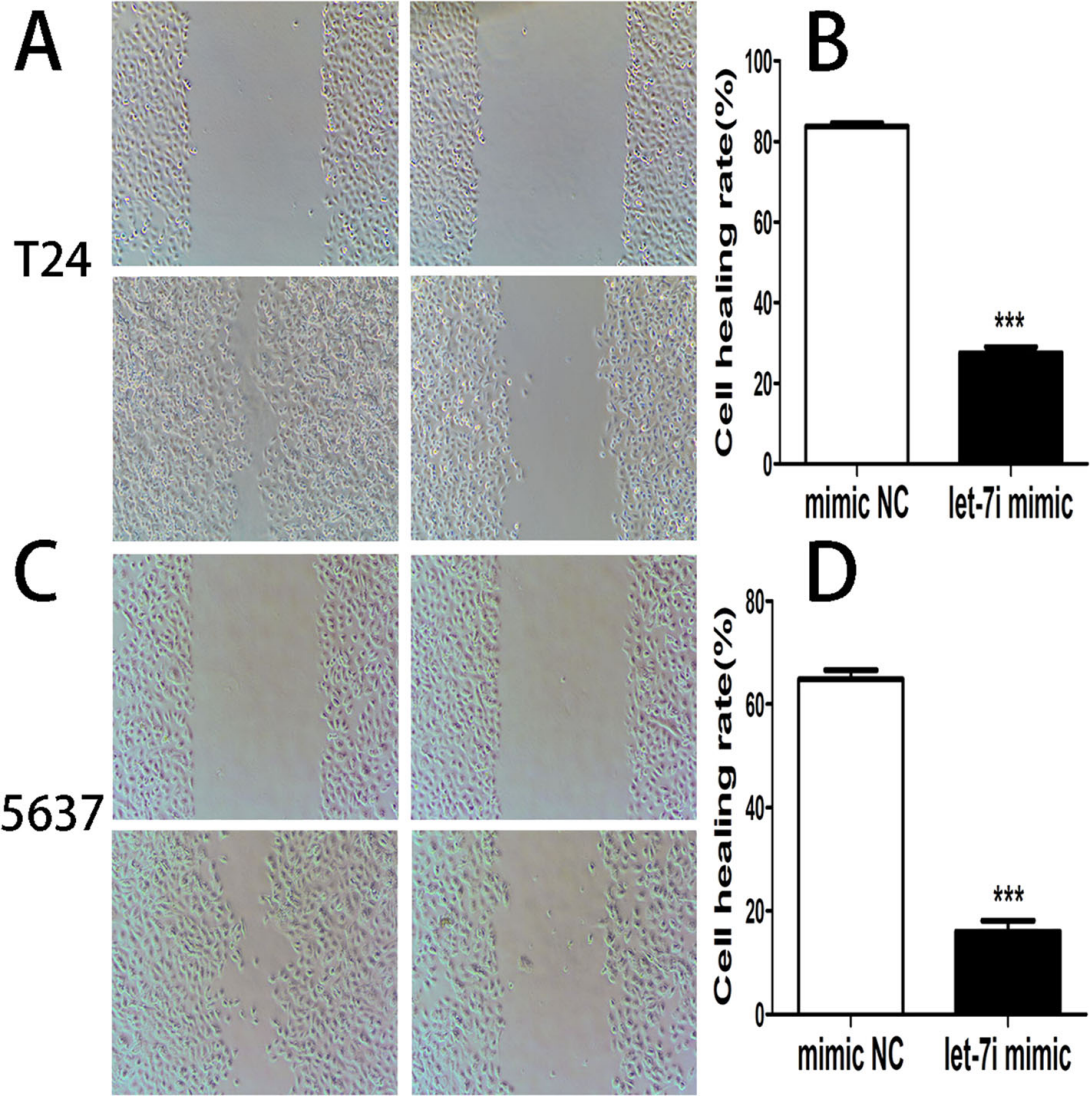
(**a**) and (**b**) The lateral migration of cell lines T24 and 5637 transfected with let-7i mimic and negative control was examined by the cell scratch assay. (**c**) and (**d**) The graph represents the mean of wound closure rates ± SEM in cell lines T24 and 5637 transfected with let-7i mimic and negative control. (***P < 0.001)

Compared with the cells transfected with negative control, the cell healing rate in cell lines T24 with let-7i mimic was downregulated, and the ability to lateral migration in cell lines 5637 with let-7i mimic was also downregulated (Fig. 4).

**Fig. 5:**
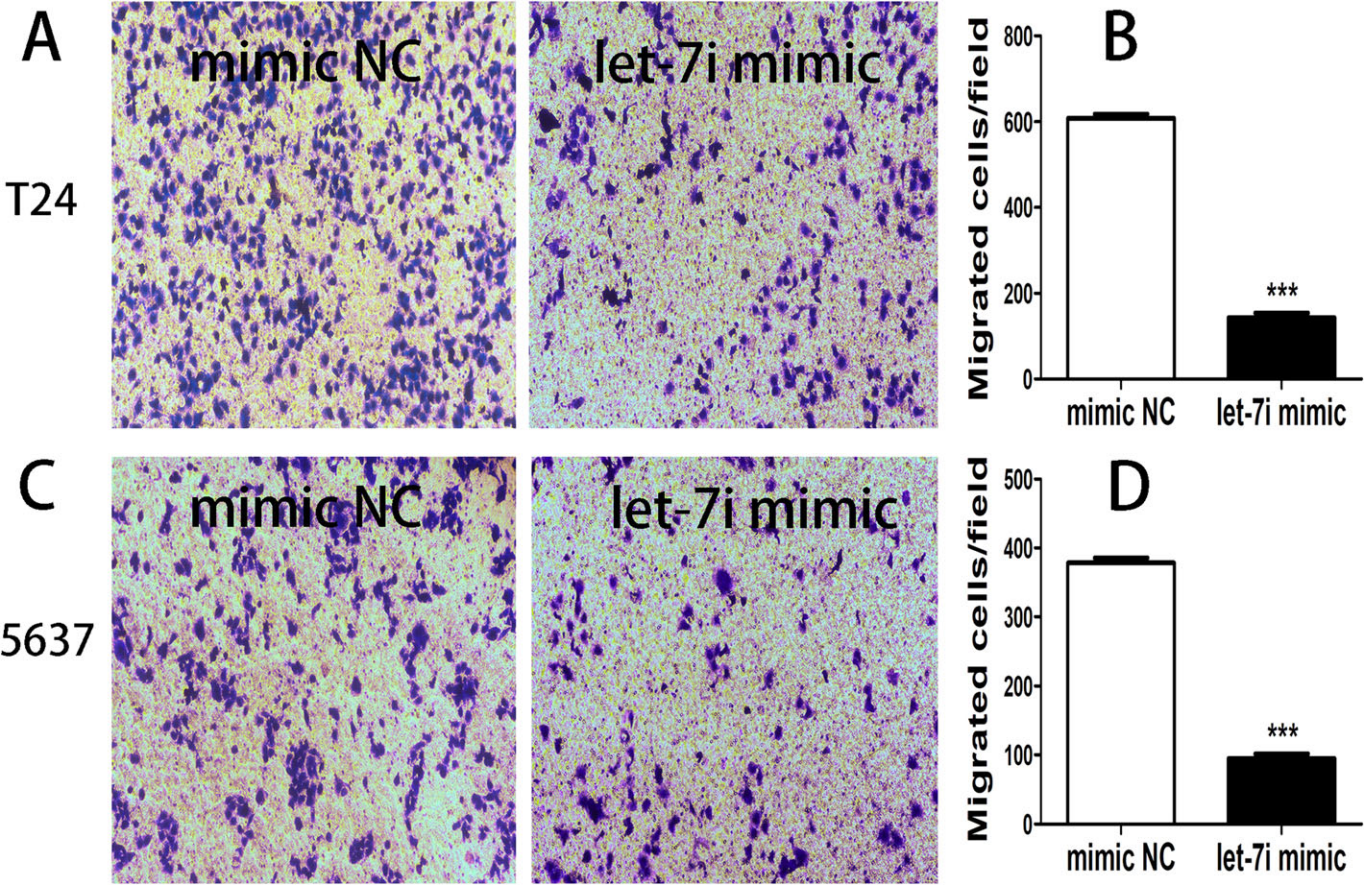
(**a**) and (**b**) The vertical migration of cell lines T24 and 5637 transfected with let-7i mimic and negative control was examined by the transwell assay. (**c**) and (**d**) The graph represents the mean of migrated cells ± SEM in cell lines T24 and 5637 transfected with let-7i mimic and negative control. (***P < 0.001)

Compared with the cells transfected with negative control, the migrated cells number in bladder cancer cell lines T24 with let-7i mimic was decreased, and the ability to vertical migration in bladder cancer cell lines 5637 with let-7i mimic was also downregulated (Fig. 5).

### HMGA1 was up-regulated in bladder cancer cell lines

**Fig. 6:**
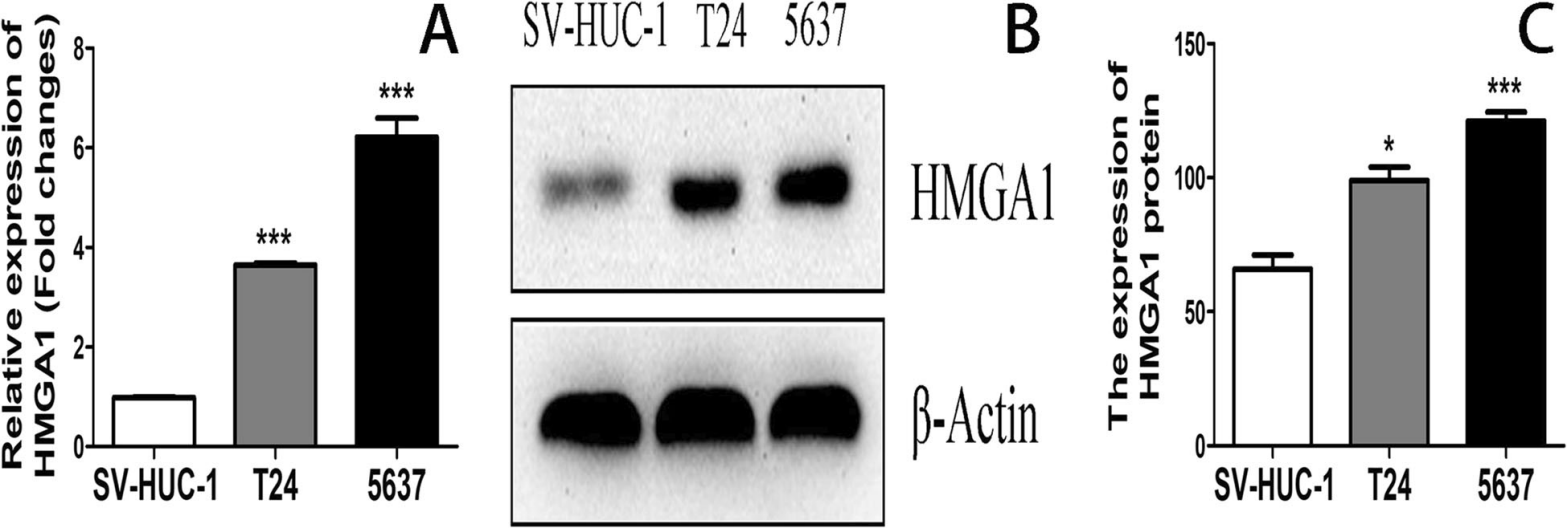
(**a**) HMGA1 mRNA expression levels in cell lines T24, 5637 and SV-HUC-1 were measured by RT-PCR. (**b**) and (**c**) HMGA1 protein expression levels in cell lines T24, 5637 and SV-HUC-1 were measured by western blotting. (*P < 0.05, ***P < 0.001)

High expression of HMGA1 mRNA was found in bladder cancer cell lines T24 (3.65 ± 0.04) and 5637 (6.22 ± 0.38) as compared to SV-HUC-1 (0.99 ± 0.01, *P* < 0.001). (Fig. 6a). Compared to SV-HUC-1, HMGA1 protein was also up-regulated in bladder cancer cell lines T24 and 5637. (Fig. 6 b and c).

### HMGA1 was a target gene of let-7i

**Fig. 7:**
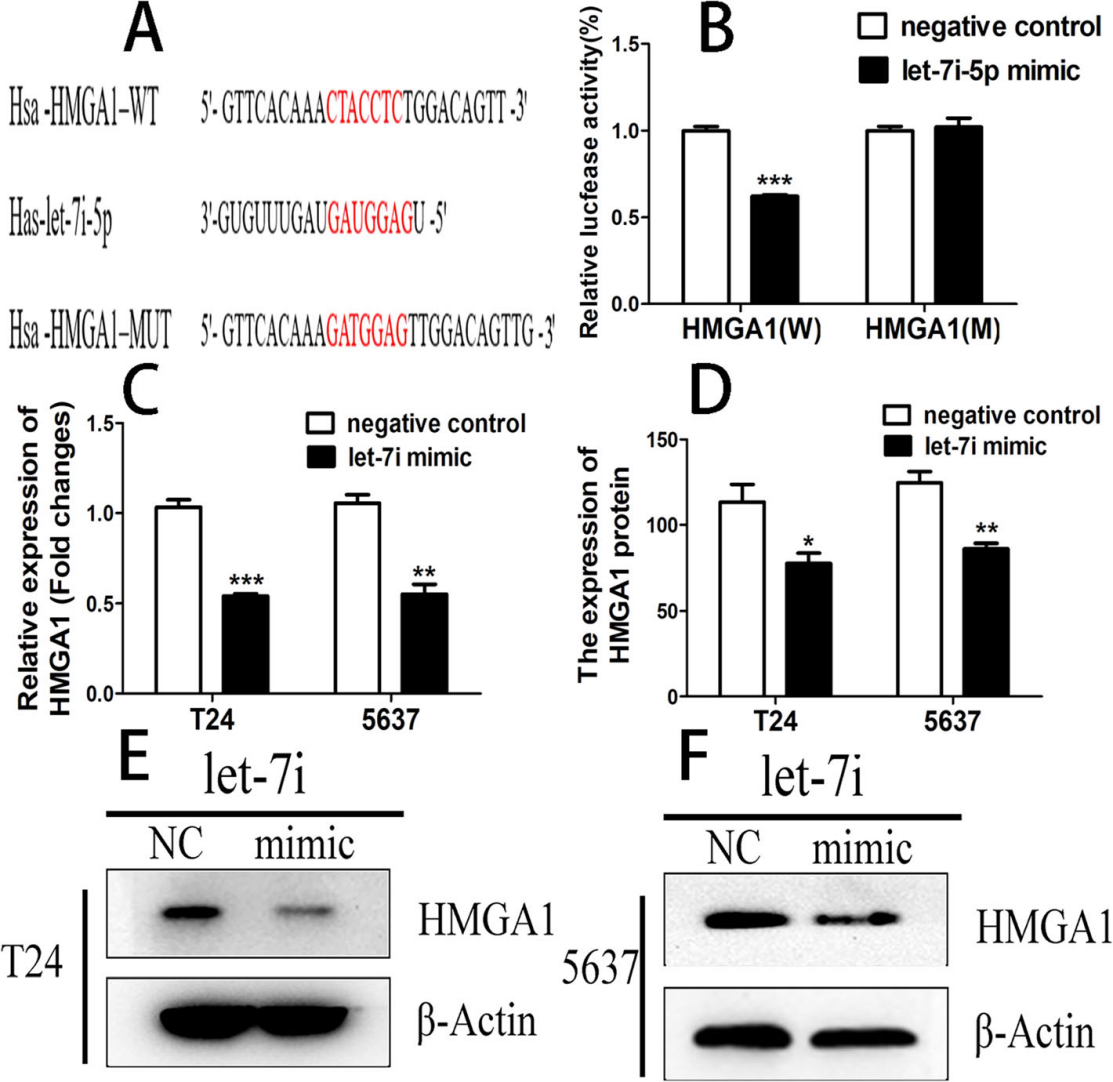
(**a**) and (**b**) Luciferase activity in the HMGA1–3′-UTR-WT group was significantly decreased after transfection with let-7i-5p mimic; (**c**) HMGA1 mRNA expression in cell lines T24 and 5637 transfected with let-7i mimic and negative control was analyzed by RT-PCR. (**d**), (**e**) and (**f**) HMGA1 protein expression levels in cell lines T24 and 5637 transfected with let-7i-5p mimic and negative control was measured by western blotting. (*P < 0.05, **P < 0.01, ***P < 0.001)

Let-7i targets were analyzed by using the bioinformatics software prediction (http://www.targetscan.com). Software analysis revealed that HMGA1 might be a potential target of let-7i based on putative target sequences of the HMGA1 3′ UTR (Fig. 7a). Luciferase assay showed that let-7i decreased the luciferase activity of the HMGA1 3′ UTR (Fig. 7b). Over-expression of let-7i obviously downregulated the mRNA level of HMGA1. (Fig. 7c). Over-expression of let-7i also obviously downregulated the protein level of HMGA1. (Fig. 7d, e and f).

## Discussion

In recent years, various studies support a role for miRNA in the origination and progression of human cancers [19, 20]. Dysregulation of miRNA activity to control both cell growth and cell metastasis may play important future roles in preventing and treating human various malignancies. Many members of let-7 family are abnormally expressed in many human tumors, such as prostate cancer [13], pancreatic cancer [21] and breast cancer [22]. Studies also showed that abnormal expression of let-7i in a variety of human tumors [23, 24], including bladder cancer [25]. However, the functions of let-7i in bladder cancer cells are unclear.

In the present study, let-7i was downregulated in bladder cancer cell lines. The results suggested that the decrease of let-7i level was correlated with the occurrence of bladder cancer. Our study was consistent with previous research [25]. Additionally, over-expression of let-7i suppressed bladder cell proliferation and migration. Song et al. found that down-regulation of the let-7i-5p inhibited the proliferation and metastasis of colon cancer cells [23, 26]. However, other study indicated that down-regulation of the let-7i facilitates gastric cancer invasion and metastasis [27]. In our study, CCK-8 and plate cloning assays showed that low level let-7i could promote the proliferation and colony formation of bladder cancer cells. Scratch and Transwell assays also indicated that the migration of bladder cancer cells was significantly inhibited after over-expression of let-7i. These results revealed that down-regulation of the let-7i could inhibit breast cancer cells proliferation and migration.

High expression levels of HMGA1 were reported in a variety of human cancers. The expression of HMGA1 is upregulate in malignant tumor derived from the prostate, breast [28, 29]. Previous reports showed that HMGA1 could affect tumor metastasis through a variety of ways [30]. In addition, Studies have shown that HMGA1 can regulate proliferation and motility of bladder cancer cells [31]. Luciferase reporter assay showed that let-7i-5p mimic could downregulate the luciferase activity of the HMGA1 WT 3′-UTR construct but not MUT 3′-UTR construct. Transfection of let-7i-5p mimic reduced HMGA1 mRNA and protein expression in bladder cancer cell lines, suggested that HMGA1 as a target gene for let-7i-5p. These results further confirmed let-7i regulated HMGA1 in bladder cancer. Increasing studies showed that the expression of let-7 in the tumor was downregulated and can inhibit the gene expression of HMGA2 and MYC at the transcriptional level [32]. Some study also showed that miR-625 and let-7i could suppresses cell proliferation and migration by targeting HMGA1 in breast cancer [18, 33]. Our study was consistent with previous reports.

However, there were also some limitations for our study. First, because levels are non-physiologic, the interpretation of the fluorescent reporter assay using co-transfection of let-7i and HMGA1 should be interpreted with caution. Second, there is no manipulating HMGA1 and reversing the effects of let-7i involved in this study. In addition, this study also lacks animal in vivo experiments, so more convincing experiments need to be further discussed in the future.

## Conclusions

In conclusion, let-7i was down-regulated in bladder cancer cells and these in vitro studies showed that up-regulation of let-7i suppressed human bladder cancer cell proliferation and migration by targeting HMGA1. Our findings justify further studying let-7i as a potential clinical diagnostic or predictive biomarker and new target for molecular therapy for human bladder cancer.

## Data Availability

The datasets used and/or analyzed during the current study available from the corresponding author on reasonable request.

## Abbreviations

3’UTRs: 3′ untranslated regions
CCK8: Cell Counting Kit-8
HMGA1: High mobility group protein A1
NMIBC: non-muscle invasive
SV-HUC-1: SV-40 immortalized human uroepithelial cell line
T24 and 5637: bladder cancer cell lines
TUR: endoscopic transurethral resection
